# Antimicrobial activity of Epsilon-Poly-l-lysine against phytopathogenic bacteria

**DOI:** 10.1038/s41598-020-68262-1

**Published:** 2020-07-09

**Authors:** Bárbara Rodrigues, Tâmara P. Morais, Paulo A. Zaini, Cássio S. Campos, Hebréia O. Almeida-Souza, Abhaya M. Dandekar, Rafael Nascimento, Luiz R. Goulart

**Affiliations:** 1 Laboratory of Nanobiotechnology, Institute of Biotechnology, Federal University of Uberlândia, Uberlândia, Brazil; 2 Federal Institute of Education, Science and Technology of South of Minas Gerais, Machado, Brazil; 3 Department of Plant Sciences, University of California, Davis, Davis, CA USA

**Keywords:** Biotechnology, Microbiology, Plant sciences

## Abstract

Antimicrobial peptides (AMPs) are components of immune defense in many organisms, including plants. They combat pathogens due to their antiviral, antifungal and antibacterial properties, and are considered potential therapeutic agents. An example of AMP is Epsilon-Poly-l-lysine (EPL), a polypeptide formed by ~ 25 lysine residues with known antimicrobial activity against several human microbial pathogens. EPL presents some advantages such as good water solubility, thermal stability, biodegradability, and low toxicity, being a candidate for the control of phytopathogens. Our aim was to evaluate the antimicrobial activity of EPL against four phytobacterial species spanning different classes within the Gram-negative phylum Proteobacteria: *Agrobacterium tumefaciens* (syn. *Rhizobium radiobacter*), *Ralstonia solanacearum*, *Xanthomonas citri* subsp. *citri* (*X. citri*), and *Xanthomonas euvesicatoria*. The minimum inhibitory concentration (MIC) of the peptide ranged from 80 μg/ml for *X. citri* to 600 μg/ml for *R. solanacearum* and *X. euvesicatoria*. Two hours of MIC exposure led to pathogen death due to cell lysis and was enough for pathogen clearance. The protective and curative effects of EPL were demonstrated on tomato plants inoculated with *X. euvesicatoria*. Plants showed less disease severity when sprayed with EPL solution, making it a promising natural product for the control of plant diseases caused by diverse Proteobacteria.

## Introduction

Phytobacteria constitute an important group of plant pathogens that reduce yields of valuable crops. They are easily disseminated and can spread quickly, causing severe bacterial infections that are difficult to control^1^. Globally, the estimate is that 20–30% of crops are lost annually due to plant diseases^2^. Proteobacteria is a diverse group comprising many important phytopathogenic bacteria that negatively impact agricultural production worldwide^3^. In terms of scientific and economic importance, *Ralstonia solanacearum*, *Agrobacterium tumefaciens* and *Xanthomonas axonopodis* pathovars are considered the second, third and sixth most relevant phytobacteria, respectively^4,5^. *R. solanacearum* is the causative agent of bacterial wilt, the main vascular disease of bacterial etiology in the world, and one of the most destructive diseases for over 200 plant species. Yield losses can reach 90% in tomato and potato crops, and 30% in tobacco^5,6^. *A. tumefaciens* (updated scientific name *Rhizobium radiobacter*) is responsible for causing crown gall, one of the most important plant diseases of grape, cherry, walnut, woody and herbaceous perennials, as well as roses and other ornamentals^7–9^. *Xanthomonas citri* subsp. *citri* is also an important pathogen that causes citrus canker, one of the most severe diseases of all commercially important citrus varieties leading to enormous yield loss, increasing production costs due to control and prevention^10–12^. *Xanthomonas euvesicatoria* is the causal agent of bacterial spot and can reduce the yield of tomato and pepper up to 50%, through reduced photosynthetic capacity and defoliation^13,14^.

When disease-resistant commercial cultivars are not available, application of chemical control is commonly used but with limited efficacy and potential environmental and human health hazards. Crop rotation can suppress some diseases, but is not always feasible and some pathogens can survive for many years in the soil or unnoticed in asymptomatic hosts. This scenario warrants an urgent need for agents that effectively control phytobacteria without selecting for resistance such as when conventional antibiotics are used. Antimicrobial peptides (AMPs) are efficient against a wide variety of pathogens due to their properties and are gaining increasing attention^15–17^. The main mechanism of action of AMPs occurs through electrostatic interactions with the anionic phospholipids of the lipopolysaccharide layer in cell membranes, compromising their physical integrity, and causing the extravasation of cellular content^18^.

Epsilon-Poly-l-lysine (EPL) is a linear homopolypeptide, generally composed by 25 to 35 identical l-lysine residues with a unique structure characterized by the peptidic bond of lysine monomers to gamma-amino functional groups and carboxyl groups^19^. The homopolymer is biodegradable, water soluble, non-toxic and highly stable at high temperatures. EPL has wide antimicrobial activity, dependent on the molecular weight of the peptide^20^. Its application against several animal pathogens is well-documented^21–25^ and it has been used as a food preservative since 1980 and considered safe for human consumption^26^. However, there is a lack of studies that demonstrate its efficacy against phytopathogenic bacteria and the possible control of plant diseases by topical application. The aim of this work was to investigate the in vitro antimicrobial potential of EPL against the phytopathogens *R. solanacearum*, *X. euvesicatoria*, *X. citri* and *A. tumefaciens* and to verify the in vivo action in the control of bacterial spot on tomato plants.

## Results

### EPL minimum inhibitory concentration and bacterial clearance dynamics

**Figure 1:**
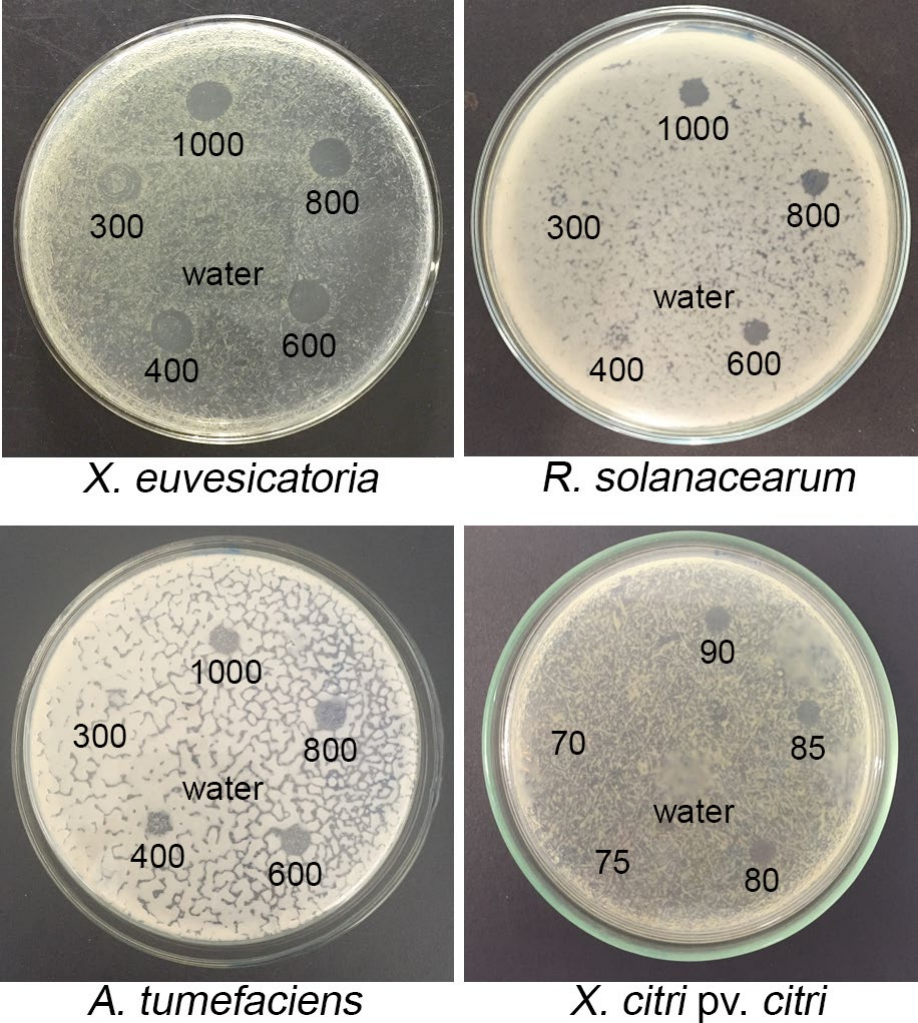
Inhibition of bacterial growth by spotting assay. Each bacterium was grown on LB agar plates and different concentrations of the peptide (70–1,000 μg/ml) were tested in different spots to determine the minimum inhibitory concentration of EPL able to inhibit bacterial growth. Water was used as negative control. The plates were placed at 28 °C for two days and evaluated. Three biological replicates were done.

**Figure 2:**
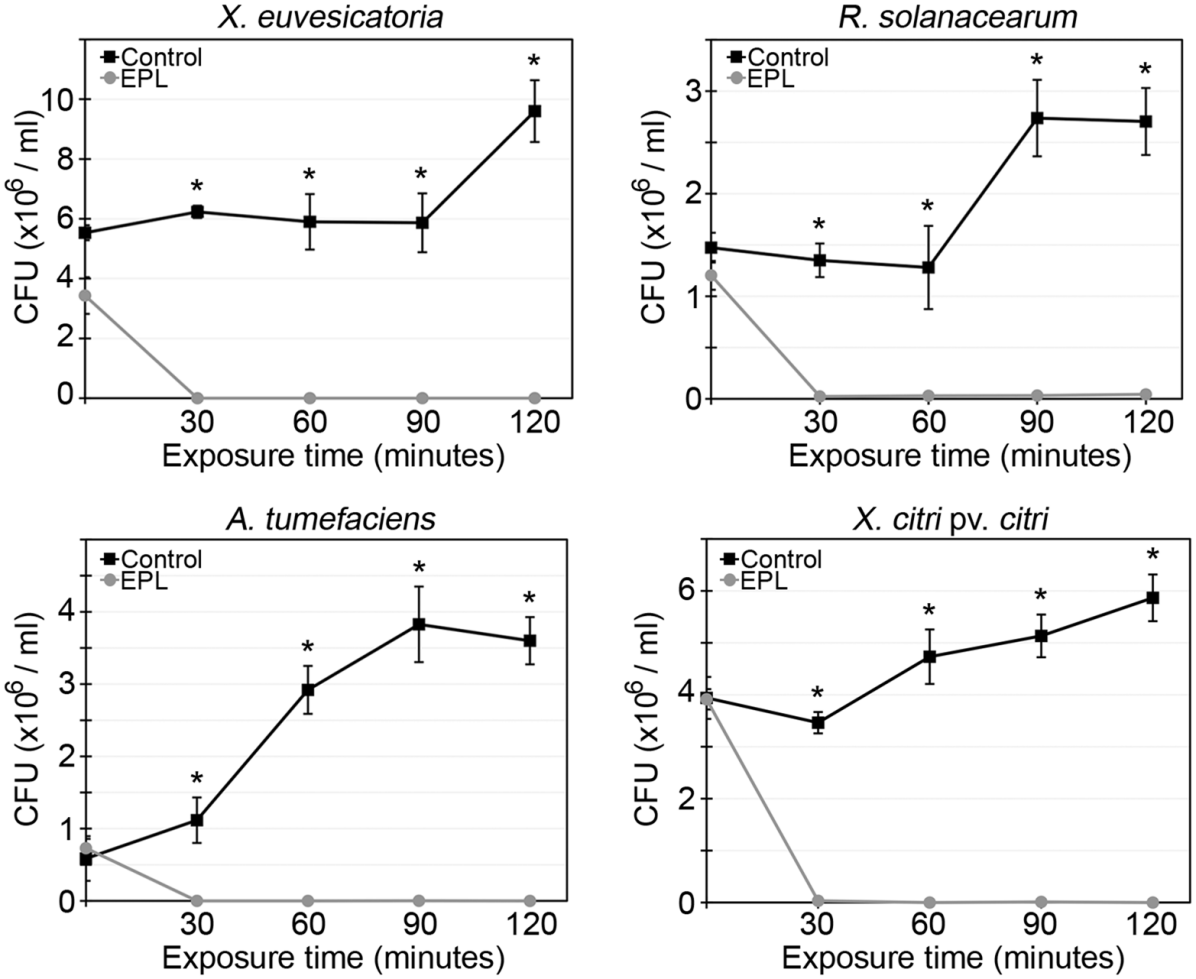
Growth inhibition curves in the presence of EPL treatment at MIC. Aliquots were taken in 30-min intervals, during 2 h, serially diluted and plated. Plates were kept for two days at 28 °C when CFU were counted. The numbers of CFU/ml are the averages for three replicates plating from each sample, and the error bars represent the standard deviations. Percentage of cell mortality was calculated as the ratio of cell counts in the treated group with EPL to those in the control group.

The antimicrobial effect of EPL was first evaluated through in vitro spotting assays*.* The minimum inhibitory concentration (MIC) was determined based on the lowest concentration of the peptide solution that was able to inhibit any bacterial growth (Fig. 1). EPL efficiently inhibited the growth of all four tested bacterial species. The MIC of EPL varied between 80 μg/ml to inhibit *X. citri* growth, 400 μg/ml for *A. tumefaciens* and 600 μg/ml for *R. solanacearum* and *X. euvesicatoria.* In order to assess a putative bactericidal effect, further investigation was conducted. Incubating the cells with the determined MICs of EPL confirmed the antibacterial activity of the peptide in the first 30 min, when the quantity of colony forming units (CFUs) in the treatment group was already statistically different from the water-treated control (Fig. 2). The peptide efficiently inhibited the growth of colonies after 30 min of EPL treatment with a reduction in bacterial growth close to 100%. After two hours, the reduction remained, confirming the bactericidal activity of EPL (Fig. 2).

### The effect of EPL on cell surface integrity and on cell viability of phytopathogenic bacteria

**Figure 3:**
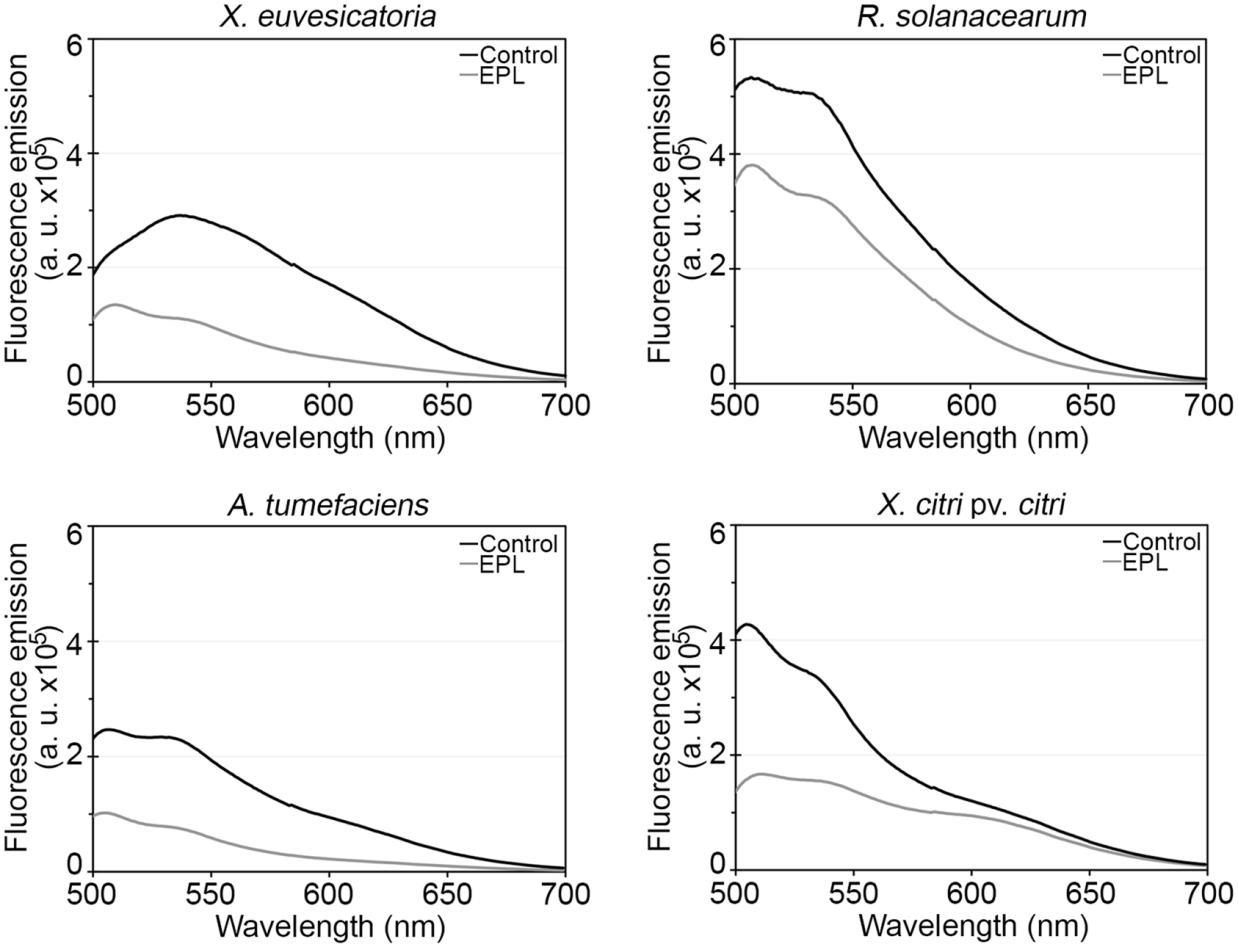
Cell viability measured by fluorescence emitted by bacterial cells treated with EPL at MIC for one hour. SYTO 9 was added to the bacterial suspensions and incubated for 15 min in the dark. Samples were excited at 470 nm and emission spectrum (490–700 nm) was recorded. A lower emitted fluorescence near 530 nm indicates a lower number of viable cells. Controls included water-only treatment (non EPL-treated) and EPL with fluorophore (without bacterial cells). Values shown are the average of three biological replicates.

**Figure 4:**
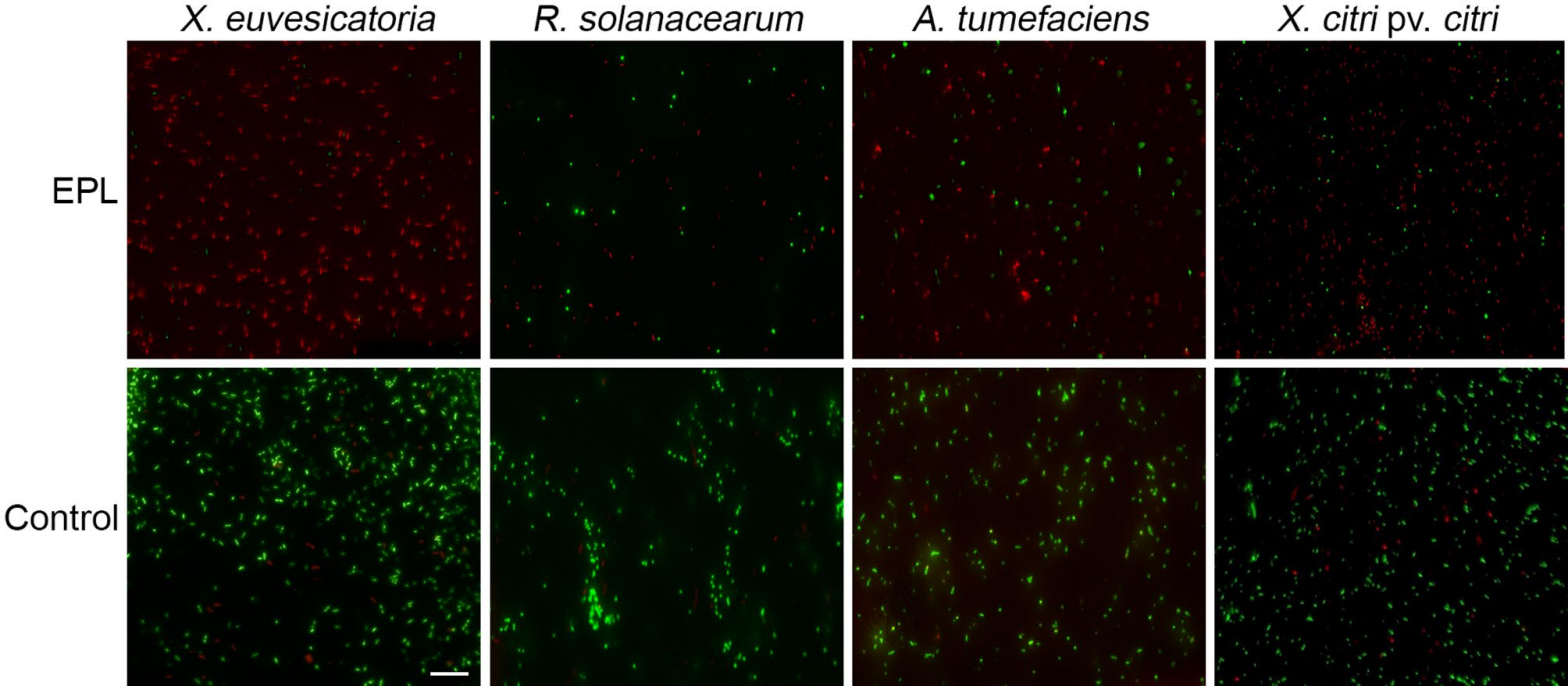
Overlap of fluorescence images of bacterial cells stained with SYTO 9 and propidium iodide for 15 min in the dark. Bacterial cells were treated with EPL at MIC for one hour or water as control. The red fluorescence is emitted because of an interaction between propidium iodide and DNA when the cytoplasmic membrane is damaged, whereas bacteria with intact cell membranes are impermeable to propidium iodide and display green fluorescence. Images shown are representative of three biological replicates. Scale bar = 10 μm.

After confirming the growth inhibition effect of EPL on all four bacterial species, we further investigated its effect on cell integrity to better understand if there was a bactericidal effect or simply growth suppression (bacteriostatic effect). To assess this, we used spectroscopy and microscopy methods. First, we measured the EPL effect on the fluorescent signal coming from cells treated with SYTO 9, a dye that binds DNA (Fig. 3). In all four bacterial species the fluorescence emission was lower in cells incubated with MIC levels of EPL compared to controls treated with water. This reduction indicates EPL is effective disrupting the cells instead of simply halting their multiplication. Next, an additional dye was used, propidium iodide, which only enters cells with damaged membranes. The combination of both dyes allows for a direct visual inspection of EPL effects, as shown in Fig. 4.

Fluorescence microscopy images showed that cells emitted red fluorescence due to interaction of propidium iodide with DNA. In the control group without EPL, the number of cells that emitted green fluorescence from intact membranes was considerably higher (Fig. 4). This result suggested that EPL-treated cells had their cell membrane damaged by the peptide.

**Figure 5:**
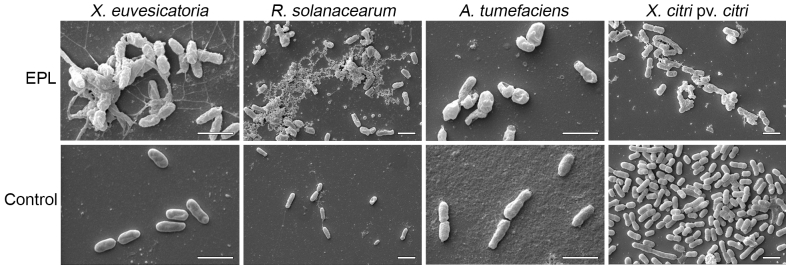
The effect of EPL on the bacterial cell surface. Scanning electron microscopy of bacterial cells treated with EPL at MIC for 1 h. No-EPL (water) treatment was used as control. Scanning electron microscope was operated at 10 kV. Scale bar = 1 μm.

To assess membrane integrity and bacterial morphology in finer detail, the bacterial cells were visualized under scanning electron microscopy (SEM) after 1 h of treatment with EPL at the MIC (Fig. 5). The SEM images revealed the rupture of the cytoplasmic membrane and the extravasation of cellular content due to the membrane integrity disruption effect of EPL. Compared with treated cells, the surface of untreated cells was bright and smooth. Taken together these results provide further support that EPL is actively damaging the bacterial cells and not just simply arresting their proliferation.

### EPL protects tomato plants against bacterial spot disease

After attesting the in vitro antimicrobial activity of EPL, we performed in vivo assays in tomato plants infected with *X. euvesicatoria* to establish the potential of this peptide to control bacterial diseases by topical application.

**Figure 6:**
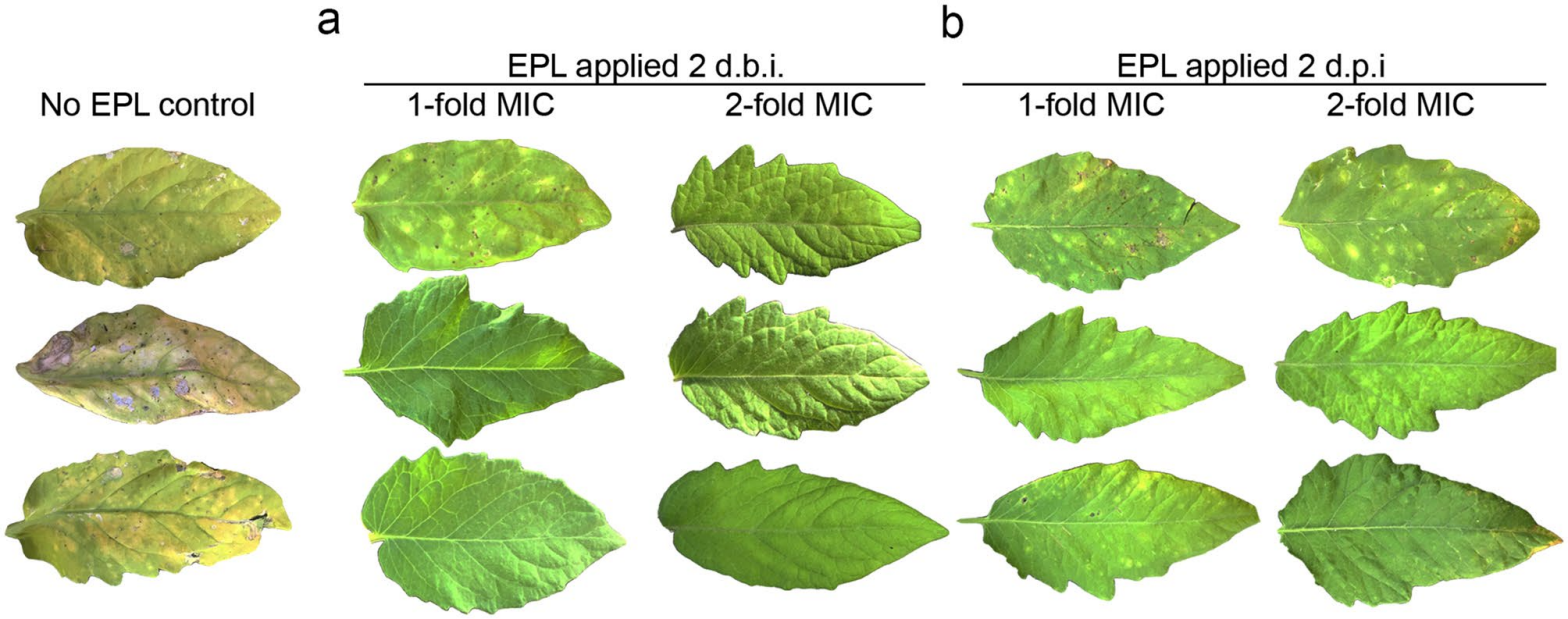
Enhanced protection against bacterial spot in tomatoes sprayed with EPL solution. Twelve Santa Cruz-Kada tomato plants were analyzed for each condition after 30 days of *X. euvesicatoria* inoculation*.* The figure shows representative leaves of three tomato plants treated with EPL (onefold MIC or twofold MIC) 2 days before inoculation (**a**), and tomato plants treated with EPL (onefold MIC or twofold MIC) 2 days post-inoculation (**b**).

Twenty days post-inoculation of the pathogen the initial symptoms such as spots and irregularly shaped watery patches on the leaves started appearing in all 12 control plants treated with water only. Three out of twelve plants that were treated preventively with EPL (at MIC level) developed initial symptoms. Interestingly, plants that were treated preventively with twofold MIC did not show any symptoms. At thirty days post- inoculation the disease was more severe in the control group (yellowish and bottom leaves death), and some plants were classified as level 5 according to the disease scale proposed by Mello et al.^27^. Plants that were treated preventively with EPL (onefold MIC) developed some disease symptoms, however no symptom was observed on plants that were treated preventively with twofold MIC, demonstrating the long-lasting effect of preventive EPL treatment (Fig. 6a).

We also tested the potential curative effect of EPL after *X. euvesicatoria* infection. Plant recovery, however, was not as robust as prevention with EPL. When EPL was applied two days after *X. euvesicatoria* infection all the plants showed initial symptoms of bacterial spot (onefold MIC and twofold MIC), however, by the end of the experiment, no plants were classified as level 5 according to the disease scale, meaning that EPL treatment can slow down the disease progress or alternatively reduce its severity (Fig. 6b).

## Discussion

Antimicrobial peptides are widespread in nature, among competing microbial communities and also among plant and animal hosts as a means to fight off infections. They hold great potential in agriculture, since phytopathogenic bacteria are still problematic to control due to lack of effective bactericides and the emergence of resistance. Here we evaluated the potential use of EPL to control some of the most devastating bacterial plant pathogens. Its effectiveness against bacterial pathogens in mammalian and safety for human consumption has been previously confirmed, making it a promising candidate for agricultural applications. A pioneering study revealed EPL to be nontoxic in a dosage level of 10,000 μg/ml and nonmutagenic in usage-relevant concentrations in a two-generation reproduction study using rats^28^. This value is 125-fold higher than what we determined here to be necessary to control *X. citri*, 25-fold higher than necessary to control *A. tumefaciens*, and around 17-fold higher than the MIC to control *R. solanacearum* and *X. euvesicatoria*, again reinforcing its safety at effective levels for bacterial clearance. Zhao et al. studied the effect of EPL and Epsilon-caprolactone (CPL) copolymer-based nanoparticles on mammalian cells viability using human breast carcinoma and human umbilical vein endothelial cells as models, and reported no apparent cytotoxic effects for both cell types even at the concentration of 1,000 μg/ml^29^, but studies on acute toxicity are also required before recommendation for commercial application. Some countries including Japan, South Korea and Canada have already regulated EPL use as a natural preservative for the food industry^19,30^. In 2004, the US Food and Drug Administration has given EPL GRAS status (generally recognized as safe), and approved its use as an antimicrobial agent in cooked or sushi rice at levels up to 50 mg/kg of rice^31^.

We demonstrated that the in vitro antibacterial activity of the peptide was able to inhibit growth of all four tested phytobacteria. These results are correlated with loss of cellular viability verified by SEM and fluorescence assays and encouraged further investigation on its therapeutic potential in vivo. Previous studies have shown great variability of microbial sensitivity to EPL, with Gram-negative bacteria consistently showing higher sensitivity compared to Gram-positive. EPL was tested against the Gram-negative *Escherichia coli* and Gram-positive *Listeria innocua*, for which the MIC was defined as 74 µg/ml for *E. coli* and 750 µg/ml for *L. innocua*^32^. To understand these differences, it is necessary to correlate the mechanism of action of the peptide, the membrane and cell wall composition of the pathogens, and the phospholipid stoichiometry of the different microbial targets^33,34^. The kill curves obtained in this study confirmed the effective and fast bactericidal effect of EPL. Our results are in accordance with other studies testing AMPs. For example, the mortality ratio of *E. coli* O157:H7 treated with different concentrations of EPL ranged from 5 to 50 µg/ml, and after 15 h was higher than 95%^25^.

Bacterial cell death is defined as the incapacity of the cell to grow as a viable colony in bacteriological media. However, there are different ways to evaluate cell viability without culturing cells. One of them is based on fluorescent probes that can be detected through fluorescence microscopy and spectroscopy^35^. Fluorescence detection is a fast and reliable technique that is valid to quantify bacteria of different genera^36^. A known effect of EPL on bacterial cells is the damage of the cell wall, compromising cellular integrity. Cell viability can be monitored using fluorescent dyes that differ in spectral characteristics and in the ability to penetrate bacterial cells. This allows reliable quantitative distinction between bacteria with intact or damaged citoplasmic membrane, consequently differentiating living and dead cells^35^. The results obtained through fluorescence spectroscopy and SEM are in accordance with the initial results that showed reduced colony formation following incubation with EPL. The predominance of red-fluorescent cells confirms that the growth inhibition is due to the bactericidal effect of the peptide and not only a bacteriostatic effect. In order to study the effects of EPL on *S. aureus* cell membrane, Tan et al. showed that after EPL treatment there was a remarkable increase in fluorescence intensity measured by propidium iodide assay indicating an increase in cell membrane permeability^37^. When *S. aureus* cells were treated with 250 μg/ml of EPL, the cells appeared collapsed, lysed and with non-integral cell morphology^37^. Similar results were obtained after treating *E. coli* cells with 50 μg/ml of EPL for 4 h. They observed that the external membrane and cytoplasm were damaged, surrounded by cell debris and with wrinkled appearance^25^. In another study the interaction between *S. aureus* and *B. subtilis* cells with nanoparticles composed by EPL and CPL were evaluated^29^. They observed the effect of EPL treatment on cell structure by SEM and similar to our findings revealed cells broken in appearance, with rupture of cell wall and membrane, lysis of cellular content, and extravasation of cytoplasm. All these observations confirm that EPL can adsorb on the surface of the microbe membrane resulting in physical damage to the cell. The previous reports showed that EPL can affect the cell membrane permeability and compromise their viability^25,37,38^. The EPL mechanism of action assures that the microorganism does not easily develop resistance.

Despite its in vitro efficacy, it is important to understand how EPL performs in vivo under more realistic conditions encountered in the field. In this study, tomato plants that were sprayed with EPL before bacterial infection were protected against bacterial spot disease. Some spots were observed in some leaves, but the disease did not fully develop. When a higher concentration was applied (twofold MIC), no symptom was observed. Therefore, our data shows the higher effectiveness of EPL when it is applied as a preventive method at higher dosages such as twofold MIC. However, we also observed symptom reduction when EPL was applied after bacterial infection, even at MIC level.

Here we concluded that EPL, even at low concentrations, has significant in vitro antimicrobial activity against diverse phytobacteria, attesting to its broad range of activity towards microbial cells*.* It is known that EPL is biodegradable, non-toxic, resistant to thermal degradation, and possess antimicrobial activity against a wide spectrum of microorganisms. Our results confirm that EPL is a promising alternative to control phytobacteriosis prophylactically. In light of these findings, more investigations will determine the optimal method for the application of this peptide in agricultural contexts, and the effect of this peptide in the pathosystem and phytosphere microbiome in general.

## Materials and methods

### Bacterial strains

EPL antimicrobial activity was tested against the phytopathogenic bacteria *Ralstonia solanacearum* strain GMI1000, phylotype I, biovar 3, provided by Dr. Carlos Alberto Lopes, Embrapa Hortaliças, Brazil; *Xanthomonas citri* subsp. *citri* 306, obtained from Dr. Shaker Chuck Farah at University of São Paulo, Brazil; *Xanthomonas euvesicatoria* EH 2009-130 and *Agrobacterium tumefaciens* C58, obtained from Dr. Alice Maria Quezado Duval, Embrapa Hortaliças, Brazil. Bacterial stocks were kept at − 80 °C in LB broth supplemented with 50% glycerol (v/v).

### Minimum inhibitory concentration assay

To obtain the minimum inhibitory concentration (MIC) of EPL necessary to prevent bacterial growth, a spotting assay^39,40^ was performed. Briefly, bacteria were cultured in LB broth at 28 °C, 200 rpm, for 12 h. Ten microliters of these bacterial suspensions were diluted in LB to 100 μl and spread onto LB agar plates. Ten microliters of EPL solutions at different concentrations (70–1,000 μg/ml) were spotted on the agar plates that had previously received the bacterial suspensions. Plates were then incubated at 28 °C until formation of clearance zones where growth inhibition could be clearly seen. MIC values were registered as the least concentration of EPL that would inhibit bacterial growth. Distilled water was used as negative control spotted in the center of each plate. All assays were performed in biological triplicates.

### Kill-curves

After EPL MIC values were determined for each bacterial species, the growth inhibition dynamics was further evaluated. Bacteria were cultured overnight in LB broth and OD_600nm_ adjusted to 0.1 with LB medium (~ 10^6^ CFU/ml). EPL was added to each bacterial suspension at the previously determined MICs and these were incubated for two hours at 28 °C and 200 rpm. Aliquots were taken at 0, 30, 60, 90, and 120-min intervals, serially diluted with LB broth and plated. Plates were kept for two days at 28 °C and the number of CFUs was used to determine the efficiency of EPL in clearing the pathogen. Three biological replicas were performed.

### Fluorescence spectroscopy and microscopy

Bacterial viability after EPL treatment was assessed by fluorescence emitted by EPL-treated compared to non-treated bacterial cells. Initially log-phase bacterial cultures were adjusted to OD_600nm_ 0.1, and centrifuged at 10,000×*g* for 15 min. Supernatant was removed and pellets suspended in EPL solutions at MIC or distilled water control. Cells were incubated at 28 °C for one hour when 1 µl of SYTO 9 from the Live/Dead BacLight bacterial viability kit (Life Technologies) was added to 1 ml of the bacterial suspensions. The samples were then incubated in the dark for 15 min and fluorescence measured using flat bottom black plates and fluorimeter (PerkinElmer). Samples were excited at 470 nm and emission spectrum (490–700 nm) recorded. Three biological replicas were performed, and two technical replica readings of each sample were taken. Controls included water-only treatment (non EPL-treated) and EPL + fluorophores (without bacterial cells). Fluorescence microscopy was used to visualize viable and non-viable cells stained with 3 µL of a 1:1 mixture of SYTO 9 and propidium iodide from the Live/Dead BacLight kit components. Cells were prepared as described above and imaged on an Evos FL fluorescence microscope (ThermoFisher), in which viable cells fluoresce in green and non-viable cells in red.

### Scanning electron microscopy

Morphological alterations on bacterial cell surface after EPL treatment were investigated by SEM. Cells were cultured in 24-well plates containing a 12 mm round coverslip and 2 ml LB in each well, for one day. EPL was then added according to previously determined MICs, or distilled water as control, and incubated for an additional hour. Coverslips containing attached cells were then fixed with 2.5% (v/v) glutaraldehyde for 16 h at 4 °C. Next, four 10-min washes were made with 0.1 M cacodylate buffer pH 7.2, and a secondary fixation in 1% (v/v) osmium tetroxide for one hour. Another three washes in cacodylate buffer were made and samples incubated in 1% tannic acid for 30 min, followed by two quick washes in water before dehydration in increasing ethanol concentrations (50–100% [v/v]) for 10 min each step. Critical point drying was then followed by sputter coating with gold at 50 mA for 120 s before being imaged with an EVO MA Carl Zeiss (Zeiss, Germany) scanning electron microscope operating at 10 kV.

### In vivo EPL protection assay

Tomato plants (*Solanum lycopersicum* L.) cv. Santa Cruz Kada were grown in the greenhouse at 25 ± 2 °C to the V_2_ developmental stage^41^. *Xanthomonas euvesicatoria* EH 2009-130 was cultured in LB medium and adjusted to 10^7^ CFU/ml with NaCl 0.85% (w/v). The bacterial suspension was sprayed on the tomato leaves until dripping. Plants were kept in a humid chamber made with a transparent plastic bag cover for 24 h before and after inoculation to facilitate infection. To test protective and curative effect of EPL to bacterial infection, onefold and twofold MIC diluted in water plus 1% Pentra-Bark surfactant were sprayed on entire tomato plant surfaces (twelve plants for each treatment). Water was used as negative control. Applications were done two days before inoculation with *X. euvesicatoria* (prophylactic) or two days after inoculation (curative). Progress of symptom development was recorded as visual appearance of leaf spots until 30 days post-inoculation, and scores were given from 1 (1% of leaf area affected) to 5 (50% or more of leaf area affected), according to the diagrammatic scale proposed by Mello et al.^27^.

### Statistical analysis

The necessary assumptions required for the analysis of variance (ANOVA) were verified. Normality of errors and variance of homogeneity were evaluated with Shapiro–Wilk and Levene tests. Kill-curve assays were analyzed in a split-plot arrangement placing the EPL treatment and the incubation time in the main and sub plots, respectively. Complex variances were applied when significant interactions were observed. Averages of peptide treatment and incubation periods were compared by Tukey test and polynomial regression, respectively. All analyses were done considering significance of 0.05.
